# Combination of Chitosan and Essential Oils for Tomatoes Protection Against the Insect Pest *Spodoptera littoralis* (Lepidoptera: Noctuidae)

**DOI:** 10.3390/insects16070718

**Published:** 2025-07-12

**Authors:** Thomas Drozdz, Philippe Couzi, Manuel Massot, Barbara Conti, Roberta Ascrizzi, David Siaussat

**Affiliations:** 1 Institut d’Ecologie et des Sciences de l’Environnement de Paris (iEES-Paris), Sorbonne Université, CNRS, INRAe, IRD, Université Paris Créteil, Université Paris Cité, F-75005 Paris, France; thomas.drozdz@gmail.com (T.D.); philippe.couzi@inrae.fr (P.C.); manuel.massot@sorbonne-universite.fr (M.M.); 2 Department of Agriculture, Food and Environment, University of Pisa, Via del Borghetto 80, 56126 Pisa, Italy; barbara.conti@unipi.it; 3 Interdepartmental Research Centre “Nutraceuticals and Food for Health”, University of Pisa, Via del Borghetto 80, 56124 Pisa, Italy; roberta.ascrizzi@unipi.it; 4 Department of Pharmacy, University of Pisa, Via Bonanno 6, 56126 Pisa, Italy

**Keywords:** chitosan formulations, biopolymer, insect control, tomato

## Abstract

Tomatoes are one of the most popular vegetables. The high level of production in the world is often offset by numerous losses that occur during production in the field or in the post-production stages. Preservation in its fresh form is a challenge, particularly due to pest attacks on stored food. A promising natural and inexpensive solution to protect against pests is the use of chitosan (CH), which can be associated with essential oils (EOs) with repellent effects. In previous studies, some protective effects have been demonstrated using chitosan films coated with EOs. In this study, we tested CH-EOs associations on tomato fruits to evaluate their efficacy against attacks by the pest *Spodoptera littoralis* (Boisduval, 1833), taking into account parameters such as age and body mass of the larvae and the effect over time (10 days) of the treatments. Our study highlights the potential of the combination of CH and cinnamon EO as an environmentally friendly solution to protect tomatoes from *S. littoralis* attack. Here we found a repellent effect of cinnamon EO combined with CH on *S*. *littoralis* larvae, with no effect on larval attractiveness or repellence for CH alone and the four other EOs tested. The main compound in cinnamon EO, (*E*)-cinnamaldehyde, had no overall repellent effect on larvae, but had specific effects when larval age, body mass, and post-treatment time were taken into account.

## Simple Summary

Tomatoes are one of the most popular vegetables in Europe. The high level of production in the world is often offset by numerous losses that occur during production in the field or in the post-production stages. Preservation in its fresh form is a challenge, particularly due to pest attacks on stored food. A promising natural and inexpensive solution to protect against pests is the use of chitosan (CH), which can be associated with essential oils (EOs) with repellent effects. Our study highlights the potential of the combination of CH and cinnamon EO as an environmentally friendly solution to protect tomatoes from *Spodoptera littoralis* (Boisduval, 1833) attack. Here we found a repellent effect of cinnamon EO combined with CH on *S. littoralis* larvae, with no effect on larval attractiveness or repellence for CH alone and the four other EOs tested.

## 1. Introduction

Tomatoes are one of the most popular vegetables in Europe and are easy to grow in many parts of the world. According to the Food and Agriculture Organisation (FAO), total world tomato production in 2021 was 189.1 million tonnes, an increase of 4% over the previous three years [1]. Annual tomato production has reached or exceeded the million-tonne threshold in many countries. The high yield and high quality of tomato are achieved in relatively cool and dry climates, but cultivation is adaptable to a variety of weather conditions, including humid tropical climates [2,3].

The way tomatoes are consumed (fresh or cooked in sauces, soups, etc.) determines the way they are harvested and stored. Preservation in the fresh form is the most delicate, particularly due to transport, diseases or pest attacks on stored foods. In fact, the production of fruit and vegetables is subject to many constraints [4,5]. Good production and preservation depend to a large extent on the methods and means used by operators in the sector in the various countries. A high level of production is often offset by numerous losses that occur during production in the fields or in the post-production stages. For example, pest infestations in the fields account for 20–50% of losses [6,7].

Post-harvest losses can be particularly important [8,9]. Losses and waste can be observed at different stages of the value chain: during storage, processing and packaging, transport and distribution, wholesale and retail markets [4,5]. The use of low temperature and humidity control is relatively effective, but is not always accessible to industry operators and does not cover all post-production stages [4]. There is still a significant hurdle to ensure good yields while offering simple, innovative and low-cost solutions adapted to the local production context.

A promising natural and renewable material is chitosan (CH), a food-grade polysaccharide composed of β-(1,4)-linked D-glucosamine and N-acetyl-D-glucosamine units. CH is produced by deacetylation from chitin, which is the second most abundant polysaccharide in existence and is the major component of fungal cell walls and arthropod exoskeletons [10]. CH is a versatile and environmentally friendly biopolymer that already has numerous applications in medicine, agriculture, food preservation and the packaging industry [10].

CH is currently authorised in the EU as an active and non-toxic basic substance for plant protection and as an environmentally friendly and safe alternative to synthetic pesticides (Regulation EC No 1107/2009) [11]. Different formulations of chitosan spray, supplemented with natural or synthetic insecticides, have been studied as pest control tools in agriculture and for the protection of food crops against pests or diseases [10].

The combination with essential oils (EOs) is a promising solution to protect vegetables against pests during production or post-harvest. Many EOs are considered safe for human consumption and pharmaceutical use. EOs with a repellent effect can help to protect foods from attacks by several insect pests. In [12], a chitosan coating film with *Ferulago campestris* EO applied to bean seeds showed a repellent effect against the seed pest *Acanthoscelides obtectus* (Coleoptera: Bruchidae), without causing adverse effects on the germination and growth of bean plants. The protective effect of CH-EOs has been demonstrated in the European Union-funded FEDKITO PRIMA project (https://fedkito.agr.unipi.it/en/home-english/ accessed on 7 July 2025), e.g., for the protection of fresh produce against *Calliphoridae* flies [11].

We studied the potential protective role of chitosan associated (or not) with EO against *Spodoptera littoralis* (Boisduval, 1833). *S. littoralis*, known as the Mediterranean cutworm, is a polyphagous pest that is particularly destructive to vegetable and fruit crops [13]. Native to Africa but well established in the South of Europe and the Mediterranean, it attacks more than 80 plant species, including tomatoes (*Solanum lycopersicum*). The caterpillars first consume the foliage, causing complete defoliation, then perforate the fruit, digging tunnels and promoting secondary rot, rendering the tomatoes unfit for consumption [13]. This phytophagous pressure makes *S. littoralis* difficult to manage; it is resistant to many classes of insecticides, which requires the implementation of integrated control strategies [14]. Few published studies have investigated this use of mixtures of EOs and CH on insect pests [10,15,16,17].

Thus, the objective of this study was to test the use of EOs combined with CH to protect tomato fruit from *S. littoralis*. We started from the hypothesis that essential oils from different plants could have repellent or attractant effects on the behaviour of larvae. Essential oils from plants were selected by the FEDKITO programme consortium on the basis of previous work and the literature showing the potential of these essential oils for this specific use. We also wanted to know whether the main compound identified in the essential oils induced the observed effects. Finally, we also aimed to conduct a detailed study of these effects, taking into account the age of the larvae and the weight of the individuals, parameters that are rarely taken into account or observed in this type of study.

## 2. Materials and Methods

### 2.1. Essential Oils Purchase and Chemical Characterisation

All essential oils used in the tests were purchased from commercial suppliers. The following essential oils were purchased from Compagnie des Sens SAS (Lyon, France), certified ‘BIO’ by Ecocert (FR-BIO-01): Cinnamon (*Cinnamomum cassia* (L.) J.Presl), Lemon (*Citrus limon* (L.) Osbeck), Mandarin (*Citrus reticulata Blanco*), Orange (*Citrus × sinensis* (L.)) *Osbeck*) and tea tree (*Melaleuca alternifolia Cheel*). The chemical characterisation was carried out at the Faculty of Pharmacy, University of Pisa, Italy. The hydrodistilled EOs were diluted to 5% in HPLC grade *n*-hexane and then injected into a GC-MS apparatus. Gas chromatography-electron impact mass spectrometry (GC-EIMS) analyses were carried out on an Agilent 7890B gas chromatograph (Agilent Technologies Inc., Santa Clara, CA, USA) equipped with an Agilent HP-5MS (Agilent Technologies Inc., Santa Clara, CA, USA) capillary column (30 m × 0.25 mm; coating thickness 0.25 μm) and an Agilent 5977B single quadrupole mass detector (Agilent Technologies Inc., Santa Clara, CA, USA). Analytical conditions were as follows: injector and transfer line temperatures 220 and 240 °C, respectively; oven temperature programmed to rise from 60 to 240 °C at 3 °C/min; carrier gas helium at 1 mL/min; injection of 1 μL (5% HPLC grade *n*-hexane solution); split ratio 1:25. Acquisition parameters were as follows: full scan; scan range: 30–300 m/z; scan time: 1.0 sec. The identification of the compounds was based on comparison of their retention times with those of authentic samples (where available), comparing their linear retention indices relative to the series of *n*-alkanes (C6-C25). Computer matching was also used against a commercial (NIST Standard Reference Database Number 69, 2014) and a laboratory-developed mass spectra library constructed from pure substances and components of commercial EOs of known composition and MS literature data [18].

### 2.2. Chitosan and Essential Oils-Enriched Chitosan Solutions

High viscosity chitosan (CH) from crab shells (molecular weight ~50,000, CAS number: 9012-76-4) was purchased from Sigma–Aldrich (St. Louis, MO, USA). For all solutions, the protocol of Peng and Li (2014) [19] was followed with minor modifications. For the 1.0% (*w/v*) pure CH solution, 1.0 g CH was dispersed in 100 mL of demineralised water containing 1.0% (*v/v*) glacial acetic acid (Carlo Erba Reagents s.r.l., Cornaredo, Italy), respectively. The solution was then stirred on a hot-plate stirrer (new type, VELP Scientifica, Usmate, Italy) at 25 °C and 7× *g* for 2 h. For the EOs-enriched CH solutions, 0.5% (*v/v*) vegetable glycerol (A.C.E.F. s.p.a, Fiorenzuola d’Arda, Italy), 0.6% (*v/v*) Tween^®^ 80 (Sigma-Aldrich, St. Louis, MO, USA) and 0.1 or 1.0% (*v/v*) of the five selected EOs were added to the previously dissolved CH. Three EO concentrations were used in this study, corresponding to 5%, 3% and 1% of the corresponding EO diluted in chitosan solution prior to application to tomatoes. The EOs-enriched CH solutions were homogenised on a hot plate stirrer at 18 °C and 28× *g* for 4 min. Glycerol is a plasticiser that improves the mechanical properties of CH, and Tween^®^ 80 is a surfactant used to ensure wettability [20]. The solutions were prepared on the same day as the experiment. The tomatoes (SAVEOL n°3422381) were completely soaked in the corresponding chitosan solution for 1 min and then left to dry for 30 min in the open air at room temperature (i.e., 19–21 °C) before being used in the behavioural tests.

### 2.3. Insects Rearing

Test insects were obtained from a laboratory strain reared on a semi-artificial diet [21] at 23 °C, 70% relative humidity and a 16:8 light/dark cycle. Animals were observed daily to allow accurate dating of larval, pupal and adult stages. The moult of the larvae was identified by the presence of the head capsule of the previous stage. Observation of this head capsule indicated that the larvae had moulted within 24 h, allowing us to perform tests on larvae of known age (comparison between one-, two- and three-day-old larvae).

### 2.4. Two-Choice Behavioural Test

**Figure 1:**
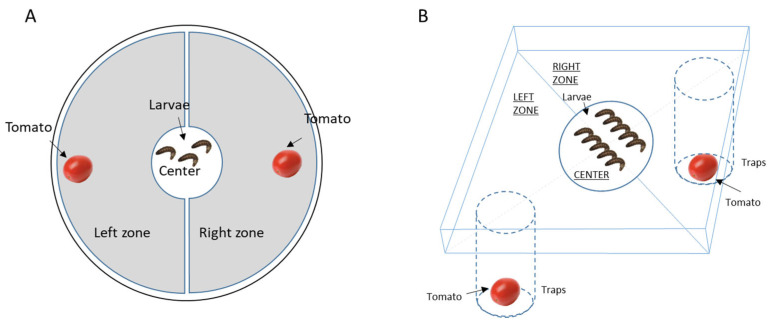
Schematic representation of the experimental design of the behavioural tests on L4 larvae in a Petri dish arena (**A**) or Pitfall Apparatus (**B**). N = 45 larvae per condition.

Behavioural tests were conducted to determine the effects of the addition of EOs to the chitosan film on larval behaviour (Figure 1A). Batches of five L4 stage (4th instar) larvae collected from our breeding stock were placed in individual boxes on the day of the experiment and placed in the dark for four hours to reduce stress until the experiment. The five larvae from the same batch were then placed in the centre of 150 mm diameter Petri dishes containing a control tomato treated with chitosan alone and a tomato treated with chitosan + EO at 5% (Figure 1A). After one hour, the number of larvae present in the centre (initial zone) and in the two zones (right or left) with the treated tomatoes was recorded. A control experiment with tomatoes treated only with chitosan was systematically carried out in parallel with these series. All tests were carried out under red light conditions (invisible spectrum for insects).

### 2.5. Tomato Consumption by Larvae

Tomatoes treated with chitosan alone or with chitosan + EO were individually placed in a Petri dish. The tomatoes were weighed before the experiment to determine their initial fresh weight. Ten L4 larvae were placed in a Petri dish. The weight of the tomatoes was recorded 48 h after the start of the experiment. We also carried out the same study on tomatoes that had not been exposed to larval attack, in order to take into account the weight loss of the tomatoes due to dehydration.

### 2.6. Behavioural Tests with Larvae of Different Ages and Weights

After a 4-h fasting period, ten L4 larvae were placed at the start of the experiment in a pitfall-type arena with a central area 2 cm in diameter (Figure 1B). At the start of the experiment, the plastic lid holding the larvae was removed, allowing the larvae to move freely within the box. Two holes were drilled in the bottom of the arena, each opening onto a closed tube, called a pitfall, in which treated or untreated tomatoes were placed. Individuals, attracted by the odours, approached the hole and had to fall into it to reach the tomato. Larvae were unable to escape from the pitfall. The control devices, which contained only tomatoes with chitosan in the branches, were also monitored in parallel to ensure random distribution between the two branches during the experiment. The experiment was carried out over 18 h (from 14.00 h on day D to 08.00 h on day D + 1). The masses of the two tomatoes and the ten L4 larvae placed in each trap were measured. For the age effect, we follow the experiment during the three days of the L4 larval instar and determine the age of larvae (i.e., one-day, two, and three-day-old L4 larvae).

### 2.7. Statistical Analyses

We analysed the two-choice tests in the arena using chi-squared tests with a Marascuilo procedure to compare the number of larvae between the arena zones (*p* < 0.05 is the level of significance). We analysed behavioural tests on aged and weighed larvae in two steps. First, we performed a logistic analysis to test whether the frequency of larvae that stayed in the experimental area (compared to larvae that were caught) depended on the age of the larvae, on the presence of chitosan, (*E*)-cinnamaldehyde or cinnamon EO, and on the interaction between these two factors. Secondly, a logistic analysis was performed on the frequency of larvae choosing the trap with the treated tomato (compared to the control tomato), with the age and body mass of the larvae as factors. For the experiment examining the persistence of the effects of the tomato coating treatments over ten days, the frequency of larvae choosing the trap with the treated tomato (compared to the control tomato) was tested using a logistic analysis with age of the larvae and time after treatment as factors. Statistical analyses were performed using JMP software (JMP Pro 16, SAS Institute Inc., Cary, NC, USA) or XLSTAT (2023 3.0 Addinsoft).

## 3. Results

### 3.1. Essential Oil (EO) Compositions

The full EO compositions are given in Table 1. A total of 84 compounds were identified among all the EOs analysed. Cinnamon (*Cinnamomum cassia* (L.) J.Presl) EO was mainly composed of phenylpropanoids (over 85%), of which (*E*)-cinnamaldehyde was detected as the most abundant compound (70.1%) in the total composition, followed by (*E*)-o-methoxycinnamaldehyde (15.2%) and (E)-cinnamyl acetate (2.1%).

Monoterpene hydrocarbons dominated the composition of all the EOs analysed from Citrus species. Within this chemical class, limonene was identified as the most abundant compound, ranging from 64.2% in lemon (*Citrus limon* (L.) *Osbeck*) to 93.7% in sweet orange EO (*Citrus × sinensis* (L.) *Osbeck*). Lemon and red mandarin EOs also contained relevant relative amounts of γ-terpinene (10.5% and 19.1%, respectively), β-pinene (10.1% and 1.4%, respectively) and α-pinene (1.4% and 1.5%, respectively). Among the Citrus species analysed, the highest relative abundance of oxygenated monoterpenes was found in lemon EO (7.5%), with trans-citral, neral and geranyl acetate being the most abundant.

More than 50% of tea tree (*Melaleuca alternifolia Cheel*) EO was composed of oxygenated monoterpenes, the most abundant of which was 4-terpineol (42.7%). Within this chemical group, α-terpineol is typically found in tea tree EO composition, as well as p-cymene and γ-terpinene, which were detected as the two most abundant monoterpene hydrocarbons (9.6% and 3.5%, respectively) [22].

### 3.2. Two-Choice Behavioural Test

**Figure 2:**
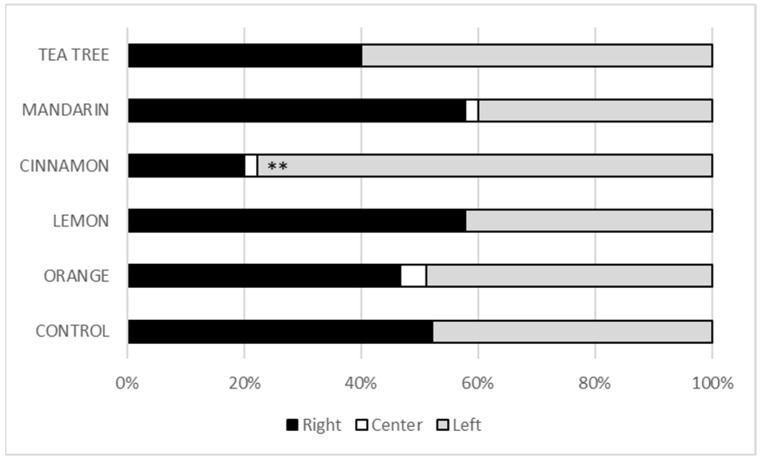
Two-choice behavioural tests. For the control experiment, two tomatoes with the chitosan film alone were placed in the right and left parts of the arena. For the experiments with EO, the tomatoes with EO at 5% were placed in the right part of the arena. Results are expressed as percentages between the three arena zones. *p*-values were greater than 5% in all experiments, except for cinnamon EO (**: *p* < 0.01). N = 45 larvae per condition.

The relevance of our two-choice experiments was supported by the observation of very few larvae remaining in the starting zone (centre zone) of the arena and the balanced proportion of larvae in the control condition, which faced two tomatoes treated with chitosan alone (Figure 2). Comparison of the different treatment conditions with tomatoes treated with chitosan alone showed no significant attraction or repellent effect of the tea tree, mandarin, lemon and orange EOs at 5%. Only cinnamon EO 5% showed a repellent effect (*p* = 0.003, Figure 2). Twenty percent of the larvae were found in the arena zone with cinnamon EO compared to 78% in the arena zone with chitosan alone.

### 3.3. Tomato Consumption by Larvae

**Figure 3:**
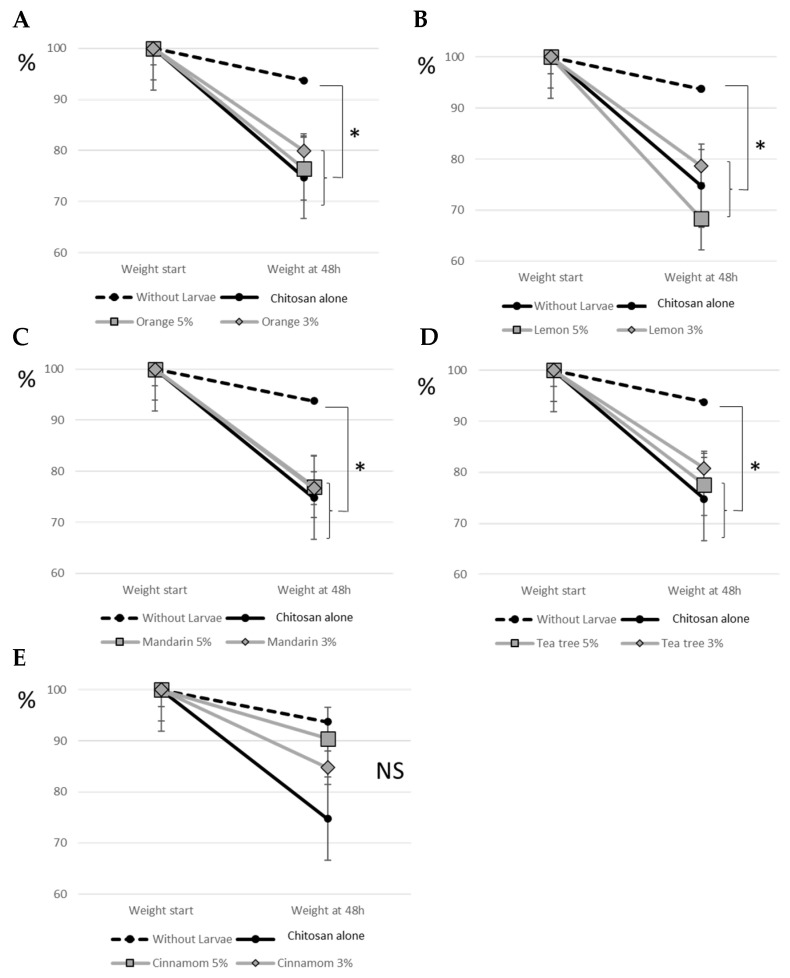
Tomato consumption by larvae after treatment by (**A**) Orange EO, (**B**) Lemon EO, (**C**) Mandarin EO, (**D**) Tea tree EO, and (**E**) Cinnamon EO. Results are expressed as a percentage of tomato weight. Standard deviations were represented for each point. The statistical analyses presented in the figure are a comparison of the results obtained in each condition with the control without larvae. *p*-values less than 5% were considered significant (*: *p* < 0.05). NS indicates no significant difference. N = 40 per condition.

To complement the previous one-hour behavioural tests, we carried out 48-h tests to investigate the effects of treatments on larval feeding behaviour. The consumption of tomatoes resulted in a loss of tomato weight and the presence of numerous bites and larval attacks. Tomato weight loss was 25.2% in the control condition with chitosan alone. Tomatoes with chitosan alone and without larvae lost 6.2% of their weight in 48 h, mainly due to dehydration. The difference between tomatoes with and without larvae was significant (*p* = 0.029) and showed the consumption of tomatoes by larvae. In comparison with the chitosan control without larvae, we also found a significant consumption of tomatoes by larvae in the conditions combining chitosan with orange, lemon, mandarin and tea tree, both for the percentages of EO of 5% and 3% (Figure 3). For cinnamon, no difference was observed with the same control without larvae, regardless of the percentage of essential oil. We measured a loss of 9.6% and 15.2% of the tomato weight for 5% and 3% cinnamon EO, respectively, whereas for the other conditions (chitosan alone or chitosan with EO), we reported losses between 20.2% and 31.7%. This means that tomato consumption by larvae was particularly reduced by cinnamon EO (Figure 3).

### 3.4. Behavioural Tests with Larvae of Different Ages and Weights

**Figure 4:**
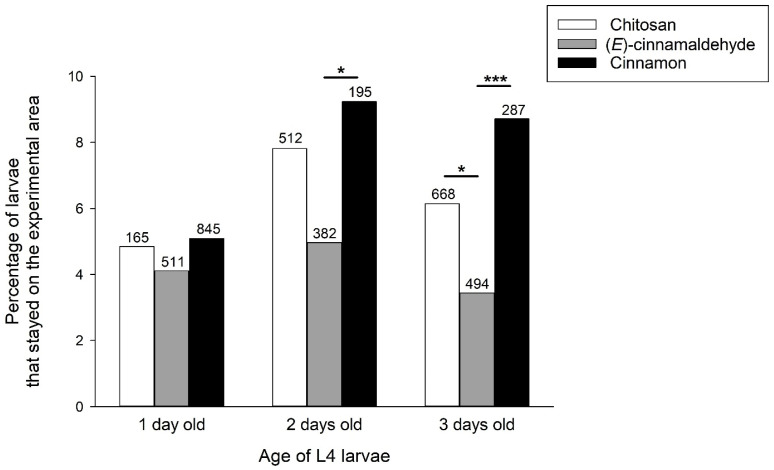
Percentage of L4 larvae that remained in the test area as a function of larval age and treatment with chitosan, (E)-cinnamaldehyde and cinnamon EO. The numbers above the bars represent the number of larvae tested. Significant post-hoc X^2^ tests comparing chitosan, (E)-cinnamaldehyde and cinnamon EO are indicated with * for *p* < 0.05 and *** for *p* < 0.001.

Additional analyses were performed with cinnamon EO and its main compound (*E*)-cinnamaldehyde to investigate the possible influence of age and body mass of L4 larvae. We first analysed the number of larvae that remained in the experimental area, i.e., larvae that were inactive or did not move into a trap with control or treated tomatoes. The number of larvae that remained in the experimental area depended on the age of the larvae (X22 = 7.5, *p* = 0.024) and the treatment of the tomatoes with chitosan, (*E*)-cinnamaldehyde or cinnamon (X22 = 11.8, *p* = 0.003). These effects were additive (X24 = 3.3, *p* = 0.506 for the interaction age × treatment). For the age effect, two- and three-day-old L4 larvae were captured less frequently than one-day-old L4 larvae (Figure 4). For the effect of tomato treatment, larvae were on average less frequently captured in the presence of cinnamon EO (with significant post hoc tests in two- and three-day L4 larvae) and more frequently captured in the presence of (*E*)-cinnamaldehyde (with a significant post hoc test in three-day L4 larvae) (Figure 4).

**Figure 5:**
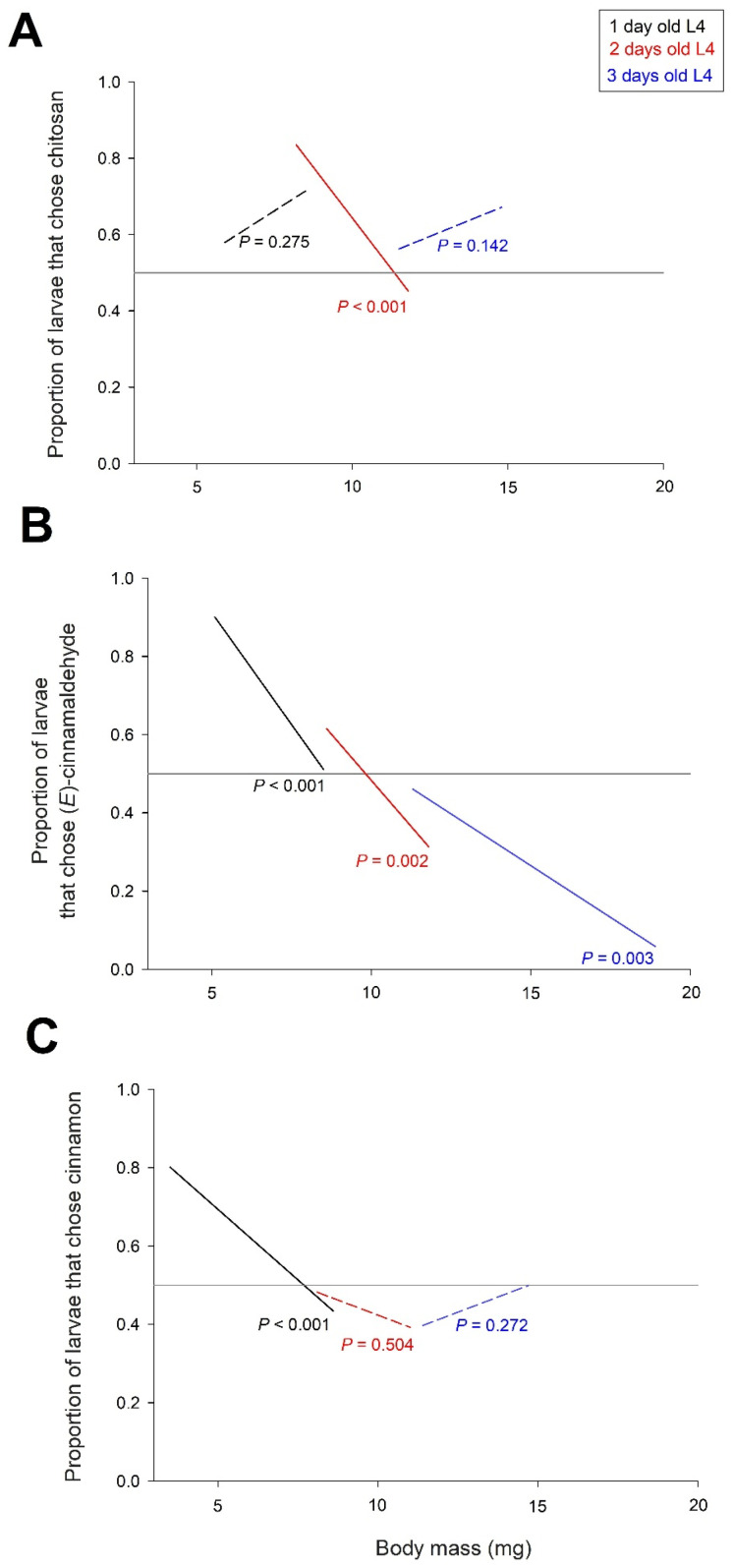
Frequency of larvae choosing the trap with the treated tomato over the control tomato as a function of age and body mass of the larvae. (**A**) Tomato treated with chitosan, (**B**) tomato treated with (E)-cinnamaldehyde, (**C**) tomato treated with cinnamon EO. The figure shows *p*-values of the relationships between the frequency of captured larvae and body mass; significant relationships with *p* < 0.05 are represented by solid lines, and non-significant relationships by dashed lines. The reference line, where the same proportion of larvae chose the treated and control tomatoes, is shown in grey. N = 165 to 845 (see Figure 4 for details).

Looking at the captured larvae, we found that their choice between the treated and control tomatoes depended on the age and body mass of the L4 larvae (Figure 5). In the chitosan/control choice experiment (Figure 5A), L4 larvae were more likely to choose the chitosan-treated tomato trap than the untreated control tomato. However, the frequency of larvae choosing the chitosan trap was dependent on the interaction between body mass and age of the larvae (X22 = 20.0, *p* < 0.001). This interaction was caused by a significant decrease in the attractiveness of chitosan with larval body mass, observed only in the two-day-old L4 larvae (Figure 5A).

In the (*E*)-cinnamaldehyde/control experiment (Figure 5B), the frequency of larvae choosing the trap with the tomato treated with (*E*)-cinnamaldehyde was dependent on body mass (X21 = 38.8 *p* < 0.001) and age of the larvae (X22 = 10.2 *p* = 0.006). The interaction between body mass and larval age was not significant (X22 = 3.8, *p* = 0.147). Figure 5B shows that the frequency of larvae choosing the (*E*)-cinnamaldehyde trap decreased with both body mass and age of the larvae. Overall, (*E*)-cinnamaldehyde was attractive in 1-day-old L4 larvae, repulsive in 3-day-old L4 larvae and with an intermediate pattern in 2-day-old L4 larvae.

In the cinnamon EO/control experiment (Figure 5C), the frequency of larvae choosing the cinnamon EO-treated tomato trap over the control tomato was dependent on the interaction between larval body mass and age (X22 = 11.6, *p* = 0.003). As shown in Figure 5C, this interaction was caused by a significant decrease in the attractiveness of cinnamon EO with larval body mass, which was only observed for 1-day-old L4 larvae. Cinnamon EO was only attractive to the smallest larvae, i.e., the smallest one-day-old L4 larvae.

**Figure 6:**
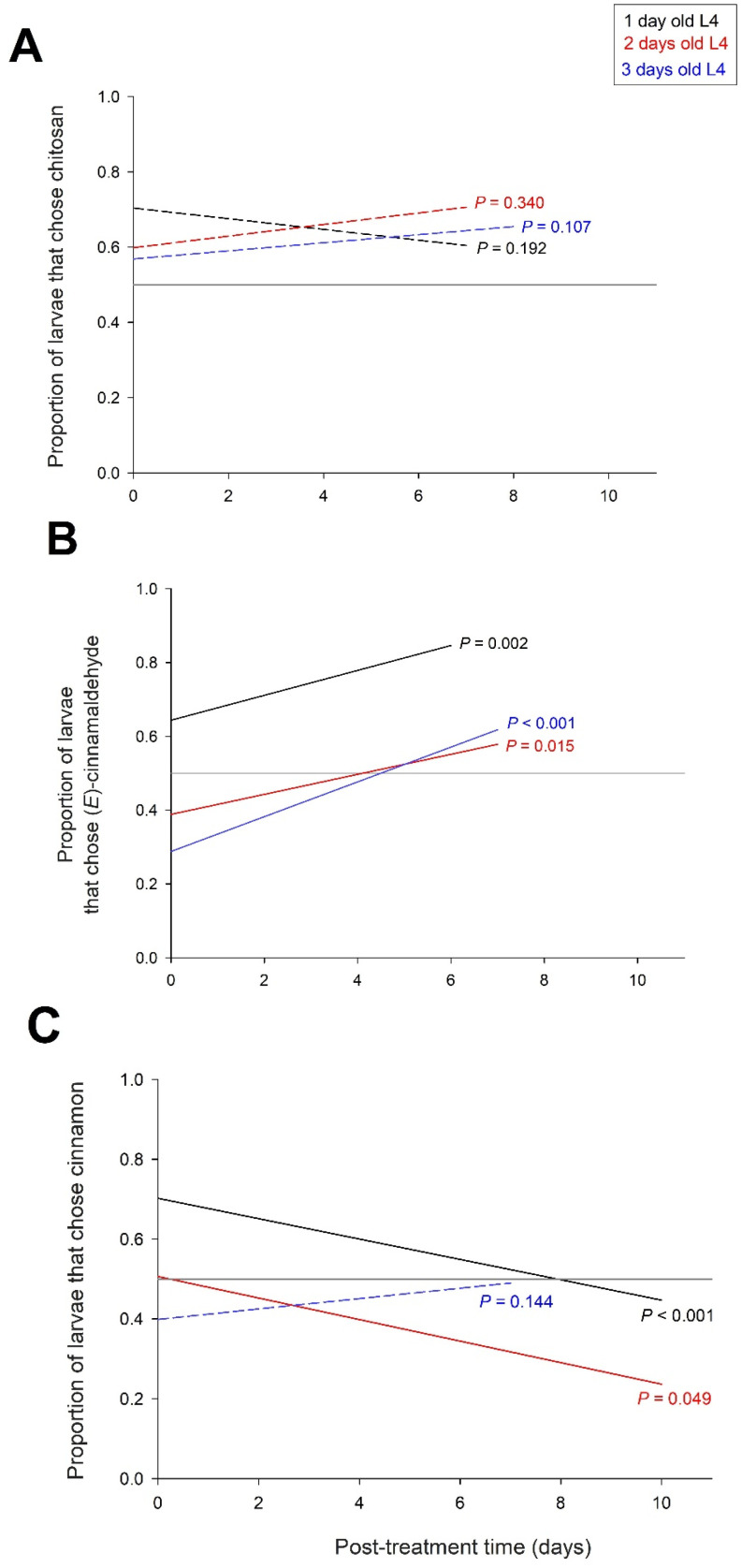
Frequency of larvae choosing the trap with the treated tomato over the control tomato as a function of larval age and time after treatment. (**A**) Tomato treated with chitosan, (**B**) tomato treated with (E)-cinnamaldehyde, (**C**) tomato treated with cinnamon EO. The figure shows the *p*-values of the relationships between the frequency of captured larvae and the post-treatment time; significant relationships with *p* < 0.05 are shown with solid lines and non-significant relationships with dashed lines. The reference line with equal proportions of larvae choosing the treated and control tomatoes is shown in grey. N = 165 to 845 (see Figure 4 for details).

In a final experiment, we tested the persistence of the effects of the treatments over a period of 10 days. The frequency of larvae that chose the trap with the tomato treated with CH over the control tomato was not significantly dependent on the time after treatment (X21 = 1.0 *p* = 0.328), the age of the larvae (X22 = 1.5 *p* = 0.476) and their interaction (X22 = 4.2 *p* = 0.122) (Figure 6A).

The frequency of larvae choosing the (*E*)-cinnamaldehyde-treated tomato trap over the control tomato trap depended on post-treatment time (X21 = 41.3 *p* < 0.001) and larval age (X22 = 121.1 *p* < 0.001), with no interaction between post-treatment time and larval age (X22 = 2.2 *p* = 0.330). The attractiveness of (*E*)-cinnamaldehyde increased over time in the three age classes of L4 larvae, and the attractiveness of (*E*)-cinnamaldehyde was higher in one-day-old larvae than in two-day and three-day larvae (Figure 6B).

The number of larvae that chose the trap with the tomato treated with cinnamon EO over the control tomato depended on the time after treatment (X21 = 5.3, *p* = 0.021), the age of the larvae (X22 = 41.5 *p* < 0.001) and their interaction (X22 = 14.4 *p* < 0.001). The frequency of larvae selecting the cinnamon EO trap decreased significantly over time in one- and two-day-old L4 larvae, but not in three-day-old L4 larvae (Figure 6C). In one-day-old L4 larvae, cinnamon EO was attractive at the beginning of the treatment but became neutral after 10 days. In two-day-old L4 larvae, cinnamon EO was neutral at the beginning of the treatment but became repellent after a few days.

## 4. Discussion

This study tested the use of EOs combined with CH to protect tomato fruit from *S. littoralis*, an important insect pest in many parts of the world [13]. Few published studies have investigated this use of mixtures of EOs and CH on insect pests [10,15,16,17]. We demonstrated a repellent effect of cinnamon EO combined with CH on *S. littoralis* larvae. No effect on larval attractiveness or repellence was found for the other four EOs tested and for CH alone. No overall repellent effect on larvae was found for (*E*)-cinnamaldehyde, but this major compound in cinnamon EO had specific effects when considering larval age and body mass, and time after treatment.

### 4.1. The Repellent Effect of Cinnamon EO

*S. littoralis* larvae were not sensitive to tea tree, mandarin, lemon and orange EO, nor to CH alone. However, cinnamon EO had a repellent effect on larvae as observed in our behavioural test (Figure 2). The consumption of tomatoes by larvae over 48 h was also reduced by cinnamon EO (Figure 3). This effect on consumption also highlighted a deterrent effect of the treatment, as larval consumption was gradually affected over the 48-h observation period. The repellent property of cinnamon EO has previously been highlighted in other insect pests [23]. More recently, the efficacy of EO from cinnamon leaves and flowers was also evaluated against two common pests, *Sitophilus oryzae* and *Callosobruchus maculatus* [24]. Leaf EO was shown to repel these pests even at low concentrations. Cinnamon EO also repels the invasive species *Cydalima perspectalis* [25], adults of *Lasioderma serricorne* [26], and has been shown to be an effective deterrent to oviposition in *Culex pipiens* [27]. Specifically, we have shown that the repellent effect of cinnamon EO is maintained when it is associated with CH. Therefore, our results suggest that the combination of CH with cinnamon EO has good potential as a biocontrol strategy against the pest moth *S. littoralis*.

### 4.2. Lack of Overall Effect of the Compound (E)-Cinnamaldehyde

EOs are complex mixtures of different molecules [17,24,27]. It has long been assumed that the effects of these complex mixtures on animal behaviour are due to their major compounds, as observed in pheromone mixtures. EOs are often described as rich in active compounds such as (*E*)-cinnamaldehyde or limonene [17,24,28]. (*E*)-cinnamaldehyde is the major compound in cinnamon EO, and limonene is the major compound in the EOs of the three Citrus species studied (Table 1). Because cinnamon EO had a repellent effect on *S. littoralis* larvae, in contrast to the studied Citrus species EOs, we tested the effect of (*E*)-cinnamaldehyde on larval feeding and behaviour. Although (*E*)-cinnamaldehyde is the major compound in cinnamon EO, we did not find a repellent effect of this molecule on larvae. Szelényi et al. [25] also showed that (*E*)-cinnamaldehyde did not induce electrophysiological responses at the antennae of the box tree moth, *Cydalima perspectalis*, and did not explain the repellent effect of cinnamon EO. The most intense electrophysiological response of the box tree moth was induced by benzaldehyde, a less abundant compound in cinnamon EO. In contrast to the box tree moth study, Deletre et al. [29] found that (*E*)-cinnamaldehyde induced a deterrent effect and an electrophysiological response at the antennae of the mosquito *Anopheles gambiae*. Thus, repellent and olfactory responses can vary between species, and the major component of cinnamon EO is not necessarily its active compound.

To explain that the major compound of EO is not necessarily its active compound, it is possible that interactions between molecules induce synergies or antagonisms. This has been raised in studies questioning the ecological importance of interactions between pheromones and volatile plant compounds, as perturbations in odorant detection have been reported with these mixtures [30]. Insect olfactory systems may have evolved to cope with such complex olfactory landscapes [31], but this remains poorly understood [32]. Potential synergies and antagonisms between molecules increase the importance of studying isolated molecules from complex mixtures. However, the single molecule approach is often studied due to the regulatory context of marketing and regulatory dossiers, which are more difficult to conduct on complex mixtures [33]. Regardless of the reason for the divergence of our results between the mixture of cinnamon EO and its main compound (*E*)-cinnamaldehyde, this divergence shows the interest in comparing their respective effects. As discussed below, our study also shows that the effects of EOs and their compounds may depend on individual variations.

### 4.3. Age- and Weight-Dependent Effects of Cinnamon EO and (E)-Cinnamaldehyde

We observed the effect of (*E*)-cinnamaldehyde when we considered the age and body mass of *S. littoralis* larvae. These effects related to ontogeny and interindividual variation are expected because age and body mass can alter species responses to toxic and olfactory substances [34,35]. Studies investigating the influence of interindividual variation on sensitivity to EOs are rare [36,37]. Guesmi et al. [37] found no difference in the repellent effect of *Anethum graveolens* EO between larvae and adults of *Tribolium confusum*, but found higher and faster mortality in larvae than adults. Pineda et al. [36] showed that *Drosophila suzukii* responded differently to Eucalyptus EO depending on sex and age-mating status by comparing newly emerged virgin adults and 5–7-day-old, mated adults. To our knowledge, no study has examined the influence of body mass on the response to EOs. Interestingly, our study showed that body mass and age of *S. littoralis* larvae influenced responses to cinnamon EO and (*E*)-cinnamaldehyde.

The attractiveness of cinnamon EO was dependent on the interaction between larval body mass and age. This interaction was caused by a decrease in the attractiveness of cinnamon EO with larval body mass, which was observed only in 1-day-old L4 larvae (Figure 5C). Cinnamon EO was only attractive to the smallest larvae. An interaction between body mass and larval age was even observed in the chitosan/control choice experiment, with a decrease in attractiveness of chitosan with larval body mass observed only in two-day-old L4 larvae (Figure 5A). The attractiveness of (*E*)-cinnamaldehyde also decreased with larval body mass. This decrease was found in the three age classes of L4 larvae studied, but resulted in contrasting attractiveness-repellence profiles. In one-day-old L4 larvae, (*E*)-cinnamaldehyde was attractive to the smallest larvae and neutral to the largest larvae (Figure 5B). In three-day-old L4 larvae, it was neutral in the smallest larvae and repulsive in the largest. An intermediate pattern around neutrality of (*E*)-cinnamaldehyde was observed in two-day-old L4 larvae.

The attractiveness of cinnamon EO and (*E*)-cinnamaldehyde decreased with larval age. They were only attractive to one-day-old L4 larvae (Figure 5 and Figure 6). This could be explained by a difference in physiology between larvae that have just moulted (one day old) and older larvae that are resuming feeding activity. An alternative hypothesis is a change in their peripheral olfactory system, as observed in *S. littoralis* larvae between their first and fourth instars [38]. Revadi et al. [38] demonstrated that this instar-specific behavioural plasticity was mediated by an odorant receptor, and therefore that the ability of insects to detect odours can change during development.

### 4.4. Time-Dependent Effect of Cinnamon EO and (E)-Cinnamaldehyde

Over 10 days, we found opposite temporal variations in attractiveness and repellence profiles between cinnamon EO and (*E*)-cinnamaldehyde. The frequency of larvae choosing tomato traps treated with cinnamon EO decreased over time in one- and two-day-old L4 larvae, resulting in the attractiveness of cinnamon at the beginning of treatment in one-day-old L4 larvae and repellence of cinnamon after 10 days in two-day-old L4 larvae (Figure 6C). In contrast, the attractiveness of (*E*)-cinnamaldehyde increased over time in the three age classes of L4 larvae studied (Figure 6B).

At the hourly scale, from post-treatment tests between 1 and 24 h, a decrease in repellence over time was found for 21 out of 28 EOS tested on the pest *Sitophilus zeamais* [39]. Cinnamon EO was tested on this pest, and the high repellence observed at 1 and 3 h after treatment disappeared after 24 h. Yang et al. [39] explained the decreased repellence of EOs over time by the high volatility of most of their low molecular weight molecules, although the volatility of EOS may also depend on the type and structure of the test surface and the EO formulation. Variation in the effects of EOs has also been described in the mosquito *Culex pipiens* [27]. Farag et al. [27] suggested that the variation in response to EOs depends on their ratios of monoterpenoids, phenylpropanoids, and fatty acids. A variation in such ratios over time could also explain the decrease in attractiveness of cinnamon EO over 10 days that we observed in one- and two-day-old L4 larvae. The variation in these ratios may have been caused by differences in the volatility of the molecules and interactions between the molecules of EO and the treated tomatoes.

## 5. Conclusions

In conclusion, our study highlights the potential of the combination of CH and cinnamon essential oil as an environmentally friendly solution to protect tomatoes from *S. littoralis* attack. However, we also show that the efficacy of essential oils may depend on the persistence of the effect over time, as well as the variation in age and body mass of the pests. Although the influence of inter-individual variation on sensitivity to essential oils is rarely considered, our results encourage studies to investigate its influence on the efficacy of new molecules for crop protection and food preservation.

To continue this study on the protection of tomatoes against *Spodoptera littoralis* using a combination of chitosan (CH) and essential oils (EO), we are considering several avenues and future work. First, we would like to investigate the effects in relation to age and during post-embryonic development. Exposures will be carried out to observe the effects at older larval stages (L5 and above) and in adults (attraction to sex pheromones). This would also allow us to test the long-term persistence of the effects. Comparative tests with other tomato pests would also be useful to see whether the treatment also has effects on other species and would provide general or specific protection. Field or storage area tests should also be planned to test this treatment in realistic conditions. We will focus particularly on the still-critical stages between the field phase and the storage phase, during which larvae can be transferred to harvested tomatoes (on or inside the tomato). The larvae can continue causing damage or even move on to other tomatoes. We will evaluate whether adding a bathing step during field harvesting can be a solution. Finally, other molecules identified in EOs would be interesting to test.

## Figures and Tables

**Table 1:** Complete chemical composition of the five studied essential oils.

Compounds	l.r.i. ^1^	Relative Abundance (%) ^2^				
		*Cinnamomum Cassia*	*Citrus limon* (L.) Osbeck	*Citrus reticulata* Blanco	*Citrus sinensis* (L.) Osbeck	*Melaleuca alternifolia* Cheel
α-thujene	931	- ^3^	0.2	0.5	-	0.3
α-pinene	941	-	1.4	1.5	0.4	1.0
benzaldehyde	959	0.4	-	-	-	-
sabinene	976	-	1.1	0.1	0.2	0.2
β-pinene	982	-	10.1	1.4	-	0.3
myrcene	993	-	1.0	0.9	1.2	-
octanal	1001	-	-	-	0.2	-
α-terpinene	1018	-	0.2	0.2	-	0.9
*p*-cymene	1027	-	0.8	0.4	-	9.6
limonene	1032	-	64.2	71.6	93.7	0.5
1,8-cineole	1034	-	-	-	-	2.5
salicylaldehyde	1047	0.2	-	-	-	-
γ-terpinene	1062	-	10.5	19.1	-	3.5
terpinolene	1088	-	0.3	0.4	-	0.8
*trans*-sabinene hydrate	1095	-	-	-	-	0.3
linalool	1101	-	-	-	0.4	-
phenylethyl alcohol	1112	0.2	-	-	-	-
*trans*-*p*-mentha-2,8-dien-1-ol	1121	-	-	-	0.4	-
*cis*-*p*-mentha-2,8-dien-1-ol	1122	-	-	-	0.5	0.2
*cis*-*p*-menth-2-en-1-ol	1124	-	-	-	-	0.3
*cis*-limonene oxide	1134	-	-	-	0.4	-
*trans*-*p*-menth-2-en-1-ol	1140	-	-	-	-	0.3
*trans*-limonene oxide	1141	-	-	-	0.4	-
hydrocinnamaldehyde	1161	0.3	-	-	-	-
borneol	1165	0.1	-	-	-	-
4-terpineol	1178	-	-	-	-	42.7
*p*-cymen-8-ol	1183	-	-	-	-	0.4
α-terpineol	1189	-	-	0.1	-	4.4
decanal	1204	-	-	-	0.5	-
(*Z*)-cinnamaldehyde	1217	0.2	-	-	-	-
*trans*-carveol	1218	-	-	-	0.6	-
*cis*-carveol	1229	-	-	-	0.2	-
neral	1240	-	1.6	-	-	-
carvone	1244	-	-	-	0.9	-
(*E*)-ascaridol glycol	1266	-	-	-	-	2.3
(*E*)-cinnamaldehyde	1268	70.1	-	-	-	-
geranial	1273	-	3.3	-	-	-
neryl acetate	1366	-	1.1	-	-	-
α-copaene	1376	1.2	-	-	-	0.2
geranyl acetate	1385	-	1.5	-	-	-
methyl N-methyl anthranilate	1406	-	-	2.6	-	-
α-gurjunene	1410	-	-	-	-	0.7
β-caryophyllene	1420	0.3	0.2	0.2	-	0.6
*trans*-α-bergamotene	1438	-	0.4	-	-	-
α-guaiene	1439	-	-	-	-	0.2
(*E*)-cinnamyl acetate	1444	2.1	-	-	-	-
aromadendrene	1445	-	-	-	-	2.6
*allo*aromadendrene	1461	0.3	-	-	-	1.3
*trans*-cadina-1(6),4-diene	1470	-	-	-	-	0.5
γ-muurolene	1477	0.4	-	-	-	-
*ar*-curcumene	1483	0.2	-	-	-	-
β-selinene	1485	-	-	-	-	0.2
δ-selinene	1490	-	-	-	-	0.4
valencene	1492	0.2	-	-	-	-
viridiflorene	1496	-	-	-	-	3.3
α-muurolene	1498	0.3	-	-	-	0.4
(*E*,*E*)-α-farnesene	1507	-	-	0.2	-	-
β-bisabolene	1509	0.3	2.1	-	-	-
*trans*-γ-cadinene	1513	0.3	-	-	-	-
(E)-*o*-methoxy cinnamaldehyde	1525	15.2	-	-	-	-
δ-cadinene	1526	0.8	-	-	-	5.0
cadina-1,4-diene	1534	-	-	-	-	0.5
α-calacorene	1546	0.3	-	-	-	-
germacrene B	1554	-	-	-	-	0.3
(*E*)-nerolidol	1565	0.3	-	-	-	-
palustrol	1568	-	-	-	-	0.3
spathulenol	1576	0.4	-	-	-	3.1
caryophyllene oxide	1581	0.5	-	-	-	-
globulol	1583	-	-	-	-	1.8
viridiflorol	1590	-	-	-	-	0.7
guaiol	1595	-	-	-	-	0.7
rosifoliol	1602	-	-	-	-	0.6
tetradecanal	1614	0.3	-	-	-	-
1-*epi*-cubenol	1628	-	-	-	-	1.0
*iso*spathulenol	1639	-	-	-	-	0.7
*epi*-α-cadinol	1641	-	-	-	-	0.7
α-bisabolol	1683	0.3	-	-	-	-
α-sinensal	1752	-	-	0.7	-	-
benzyl benzoate	1764	0.3	-	-	-	-
phenylethyl benzoate	1860	0.2	-	-	-	-
rimuene	1930	0.4	-	-	-	-
pimaradiene	1941	0.3	-	-	-	-
*m*-camphorene	1960	-	-	0.2	-	-
13-*epi*-manoyl oxide	2010	0.1	-	-	-	-
-----------------------------						
Monoterpene hydrocarbons		-	89.8	96.1	95.5	17.1
Oxygenated monoterpenes		0.1	7.5	0.1	3.8	53.3
Sesquiterpene hydrocarbons		4.6	2.7	0.3	-	16.2
Oxygenated sesquiterpenes		1.5	-	0.7	-	9.6
Diterpene hydrocarbons		0.7	-	0.2	-	-
Oxygenated diterpenes		0.1	-	-	-	-
Phenylpropanoids		87.9	-	-	-	-
Others		1.6	-	2.6	0.7	-
**Total identified (%)**		**96.5**	**100.0**	**100.0**	**100.0**	**96.2**

^1^ Linear retention index on a HP-5MS capillary column; ^2^ Detection threshold ≥0.1%; ^3^ Not detected.

## Data Availability

The original contributions presented in this study are included in the article. Further inquiries can be directed to the corresponding author.
